# Synthesis and Characterization of Macrocyclic Chiral Tröger’s Base Phenhomazine Candidates as Anticancer Agent

**DOI:** 10.3389/fchem.2020.633065

**Published:** 2021-01-28

**Authors:** Alhussein A. Ibrahim, Korany A. Ali, Naglaa A. Abdel Hafez, Mohamed A. Elsayed, Khalid M. H. Mohamed, Hanaa M. Hosni, Abd El-Galil E. Amr, Elsayed A. Elsayed

**Affiliations:** ^1^ Applied Organic Chemistry Department, National Research Centre, Giza, Egypt; ^2^ Pharmacognosy Department, National Research Centre, Giza, Egypt; ^3^ Pesticide Chemistry Department, National Research Center, Cairo, Egypt; ^4^ Pharmaceutical Chemistry Department, Drug Exploration and Development Chair (DEDC), College of Pharmacy, King Saud University, Riyadh, Saudi Arabia; ^5^ Organic Chemistry Department, Chemical Industries Research Division, National Research Centre, Cairo, Egypt; ^6^ Bioproducts Research Chair, Zoology Department, Faculty of Science, King Saud University, Riyadh, Saudi Arabia; ^7^ Chemistry of Natural and Microbial Products Department, National Research Centre, Cairo, Egypt

**Keywords:** chiral macrocyclic, tröger’s base, trögerophane, phenhomazines, anticancer activity

## Abstract

1,4,7,10-Tetraoxa[10](2,8)trögerophane **5** was synthesized from its corresponding precursors. Heating of **2** with p-nitrophenoxide afforded bis(p-nitrophenyl)ether **3**, which was treated with hydrazine hydrate to give bis(p-aminophenyl)ether **4**. Treatment of **4** with paraformaldehyde and triflouroacetic anhydride gave trögerophane **5**. Reaction of **5** with trifluroacetic anhydride afforded phenhomazine derivative **6**, which was treated with potassium carbonate to afford tetrahydrophenhomazine **7**. Finally, reaction of **7** with phenacylchloride, bromoacetic acid, or ethyl bromoacetate in the presence of triethyl amine under reflux, afforded the corresponding macrocyclic compounds **8, 9** and **10**, respectively. The synthesized trögerophane,precursors and its newly synthesized phenhomazines derivatives were screened for anticancer activity. Results revealed that 1,4,7,10-tetraoxa[10](2,8)trögerophane had a promising selectivity towards colon cancer cell line with an IC_50_ of 92.7 µg/ml.

## Introduction

**FIGURE 1:**
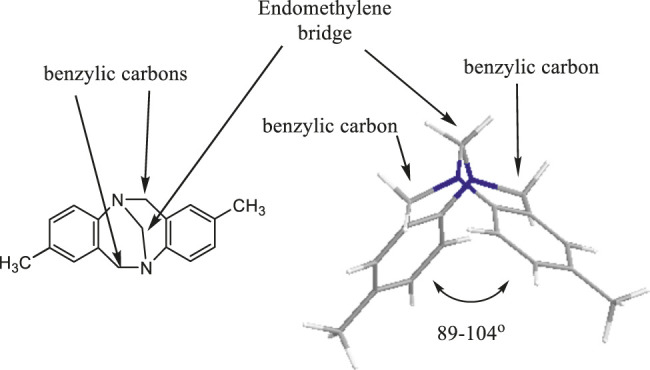
Tröger’s base structure.

Tröger’s base, “5,11-methano-2,8-dimethyl-5,6,11,12-tetrahydrodibenzo[b,f][1,5]diazocine” (Figure 1), was first prepared by Julius Tröger in 1887 by condensing dimethoxymethane with p-toluidine in the presence of hydrochloric acid (Tröger, 1887). In 1998, Alhussein et al. have reported a series of macrocyclic Tröger bases and their optical and complexing properties and named them for the first time as Trögerophanes (Ibrahim, et al., 1998; Miyahara, et al., 1999).

Tröger base and some of its macrocyclic analogues have been reported as anticancer agents (Johnson, et al., 1993; Gaslonde, et al., 2011; Paul, et al., 2012), antibacterial, antifungal and antifeedant (Thirunarayanan, 2017) and cytostatic activities (Kaplánek, et al., 2015). Although Tröger base and its analogues have attracted interest of many researcher groups due to their fascinating structures and properties (Yuan, et al., 2011), however, attention has been inadequately focused on these compounds from the biological point of view (Bailly, et al., 2000; Baldeyrou, et al., 2002; Manda, et al., 2014; Yang, et al., 2015). In addition, some of macrocyclic hetero-nitrogen derivatives have been synthesized (Abu-Ghalia, et al., 2012; Amr, et al., 2019; Naglah, et al., 2020) and have shown promising biological activity, i.e. analgesic and anticonvulsant (Amr, 2005), antimicrobial (Amr, et al., 2006; Azab, et al., 2016), anti-proliferative, 5α-reductase inhibiting (Alanazi, et al., 2020), pharmacological (Al Thagfan, et al., 2018), anticancer (Amr et al., 2018), as well as biological activities (Khayyat and Amr, 2014). In view of these observations and in continuation of our previous work in macrocyclic chemistry, we synthesized some new Trögerophane derivatives and tested their anticancer activities.

## Materials and Methods

### Materials

Triethylene glycol bis(p-aminophenyl) ether 4 was synthesized according to previously reported procedure (Ibrahim, et al., 1998). Paraformaldehyde, methanol, trifluroacetate, hydrazine hydrate, triethyl amine, phenacyl chloride, bromoacetic acid, and ethyl bromoacetate were all purchased from Sigma-Aldrich (Switzerland). All melting points were measured on a Gallenkamp melting point apparatus (Weiss Gallenkamp, London, UK). The infrared spectra were recorded in potassium bromide disks on a Pye Unicam SP 3300 and Shimadzu FT IR 8101 PC infrared spectrophotometers (Pye Unicam Ltd. Cambridge, England and Shimadzu, Tokyo, Japan, respectively). The NMR spectra were recorded on a Varian Mercury VX-300 NMR spectrometer (Varian, Palo Alto, CA, United States). 1H NMR spectra were run at 300 MHz and 13C NMR spectra were run at 75.46 MHz in deuterated chloroform (CDCl3) or dimethyl sulfoxide (DMSO-d6). Chemical shifts are given in parts per million and were related to that of the TMS as internal reference. Mass spectra were recorded on a Shimadzu GCMS-QP 1000 EX mass spectrometer (Shimadzu) at 70 eV. Elemental analyses were carried out at the Microanalytical Centre of Cairo University, Giza, Egypt and recorded on Elementar-Vario EL (Germany) automatic analyzer. TLC was performed on silica gel aluminum sheets, 60 F254 (E. Merck). Compounds 2-4 were prepared according to the reported literature (Ibrahim, et al., 1998).

### Synthesis of 1,4,7,10-tetraoxa[10](2,8)trögerophane (**5)**

To a mixture of triethylene glycol bis(p-aminophenyl) ether 4 (0.62 g, 0.0019 mol), conc. HCl (114 ml), and TFA (50 ml) in ethanol (240 ml) was stirred with cooling in an ice-bath to −10°C, paraformaldehyde (0.5 g) was added portion-wise while stirring. The reaction mixture was allowed to cool to room temperature and left for stirring at 60–70°C for further 18 h. Then, the mixture was concentrated under vacuum, basified with 28% ammonia solution, and extracted with methylene chloride (100 ml x 3). The methylene chloride phases were combined, dried (magnesium sulfate), and evaporated under vacuum to give a crude product which was purified by column chromatography (20 cm × 4 cm, silica gel 60, chloroform) to give **5** as colorless cubic crystals after recrystallization from ethanol/methylene chloride. Yield 43%, m.p. 233–235 °C (Lit. mp: 234–235 °C [2]). IR (film): ν = 3030, 3000 (C-H aromatic), 2930, 2900 (C-H aliphatic), 1605 (C-C stretching) cm−1. 1H NMR (300 MHz, CDCl_3_): δ = 2.53–2.70 (m, 4H, O-CH_2_-CH2), 3.50–3.63 (m, 4H, O-CH_2_-CH_2_), 3.98 (d, 2H, J = 16.17 Hz, -CH_2_-Ar), 4.09–4.22 (m, 4H, O-CH_2_-CH_2_), 4.46 (s, 2H, -N-CH_2_-N-), 4.57 (d, 2H, J = 16.17 Hz, -CH_2_-Ar), 6.49 (d, 2H, J = 2.97 Hz. Ar-H), 6.83 (dd, 2H, J_1_ = 5.94 Hz, J_2_ = 2.64 Hz, Ar-H), 7.01 (d, 2H, J = 8.58 Hz, Ar-H). 13C NMR (75 MHz, CDCl_3_): δ = 155.41, 141.58, 128.66, 125.07, 118.24, 116.86, 72.78, 69.88, 69.51, 68.40, 60.63 (21 C). MS (EI, 70 eV): m/z (%) = 368 (32) [M]^+^. C_21_H_24_N_2_O_4_ (368.43). Calcd: C 68.46; H 6.57; N 7.60; found: C 68.29; H 6.57; N 7.45.

### Synthesis of N-Trifluroacetamide Phenhomazine Trifluroacetate (**6)**

The trögerophane **5** (0.239 g, 0.0065 mol) in trifluroacetic anhydride (10 ml) was refluxed for 65 h, after which the unreacted anhydride was recovered using a Dean-Stark trap. To the remaining residue, ethanol (20 ml) was added and the mixture was boiled under atmospheric pressure to get rid of the least traces of the anhydride. The remaining ethanol was removed under vacuum. The resulting colorless viscous liquid was treated with diethyl ether to afford colorless needles of the desired salt **6**. Yield 92%, m.p. 150–151°C (Dec.). IR (film): ν = 1697 (C=O) cm^−1^. 1H NMR (300 MHz, CDCl_3_): δ = 7.27 (d, 1H, J = 8.91 Hz, Ar-H), 7.23–7.86 (brs, 2H, N-H, exchangeable with D_2_O), 6.94 (d, 1H, J = 8.58 Hz, Ar-H), 6.87−6.80 (m, 2H, Ar-H), 6.54 (d, 1H, J = 2.64 Hz, Ar-H), 6.44 (d, 1H, J = 2.97 Hz, Ar-H), 5.48 (d, 1H, J = 14.5 Hz, Ar-CH_2_-N), 4.84 (d, 1H, J = 13.5 Hz, Ar-CH_2_-N), 4.35–3.99 (m, 4H, O-CH_2_-CH_2_), 4.23 (d, 1H, J = 14.9 Hz, Ar-CH_2_-N), 4.22 (d, 1H, J = 13.5 Hz, Ar-CH_2_-N), 3.68–3.51 (m, 4H, O-CH_2_-CH_2_), 3.41–3.18 (m, 4H, O-CH_2_-CH_2_). MS (EI, 70 eV): m/z (%) = 566 (42) [M]^+^. C_24_H_24_N_2_O_7_F_6_ (566.45). Calcd: C 50.89; H 4.27; N 4.95; found: C 51.03; H 4.40; N 4.94.

### Synthesis of 1,4,7,10-Tetraoxa[10](2,8)-5,6,11,12-tetrahydrophenhomazine (**7)**

A mixture of trifluroacetate salt **6** (0.368 g, 0.0065 mol) and potassium carbonate (1.01 g) in methanol (15 ml) was stirred at 50 °C for 3 h. The solvent was evaporated under reduced pressure and the residue was treated with chloroform. The undissolved matter was filtered off, and the filtrate was evaporated under vacuum to give the free base as a white powder which could be recrystallized from ethyl acetate to afford 7 as colorless crystals. Yield 79%, m.p. 172–173 °C. IR (film): ν = 3397 (N-H) cm^−1^. 1H NMR (300 MHz, CDCl_3_): δ = 6.68 (d, 1H, J = 2,51 Hz, Ar-H), 6.66 (d, 1H, J = 2,51 Hz, Ar-H), 6.50 (d, 2H, J = 2,51 Hz, Ar-H), 6.49 (d, 1H, J = 2,51 Hz, Ar-H), 6.47 (s, 1H, Ar-H), 4.79 (d, 2H, J = 13,55 Hz, Ar-CH_2_-N), 4.29−4.20 (m, 2H, O-CH_2_-CH_2_), 4.08−3.96 (m, 2H, O-CH_2_-CH_2_), 3.97 (d, 2H, J = 14.05 Hz, Ar-CH_2_-N), 3.96−3.72 (br.s, 2H, N-H, exchangeable with D_2_O), 3.69−3.59 (m, 2H, O-CH_2_-CH_2_), 3.48−3.38 (m, 2H, O-CH_2_-CH_2_), 3.11−3.01 (m, 2H, O-CH_2_-CH_2_), 2.88−2.79 (m, 2H, O-CH_2_-CH_2_). 13C NMR (75 MHz, CDCl_3_): δ = 152.99, 143.18, 128.81, 120.59, 119.62, 117.14, 71.03, 69.60, 68.86, 51.84 (20 C). MS (EI, 70 eV): m/z (%) = 356 (25) [M]^+^. C_20_H_24_N_2_O_4_ (356.42). Calcd: C 67.40; H 6.79; N 7.86; found: C 67.34; H 6.85; N 7.77.

### Synthesis of N,N`-Diphenacyl Phenhomazine (**8)**

To a mixture of the tetrahydrophenhomazine **7** (0.356 g, 0.001 mol), and phenacyl chloride (0.308 g, 0.002 mol) in ethanol (100 ml), triethyl amine (1.0 ml) was added dropwisely. The reaction mixture was refluxed for 2 h. The reaction was followed up by TLC. At the end, the reaction mixture was evaporated under vacuum. The solid formed was collected and recrystallized from ethanol to give **8**. Yield 64.4%, m.p. 190–192°C. IR (film): ν = 3044, 3020, 2982, 2966, 1698, 1694 cm^−1^. 1H NMR (300 MHz, CDCl_3_): δ = 7.61−7.58 (m, 4H, Ar-H), 7.45−7.39 (m, 6H, Ar-H, 6.70 (d, 2H, J = 8,56 Hz, Ar-H), 6.60 (dd, 2H, J_1_ = 2,62 Hz, J_2_ = 8.56 Hz, Ar-H), 6.43 (d, 2H, J = 8,56 Hz, Ar-H), 5.55 (d, 2H, J = 13,90 Hz, Ar-CH_2_-N), 4.50 (s, 4H, N-CH_2_-CO-), 4.45 (d, 2H, J = 13,90 Hz, Ar-CH_2_-N), 4.28−4.20 (m, 2H, O-CH_2_-CH_2_), 4.03−3.95 (m, 2H, O-CH_2_-CH_2_), 3.62−3.44 (m, 4H, O-CH_2_-CH_2_), 3.37−3.16 (m, 2H, O-CH_2_-CH_2_), 2.99−2.82 (m, 2H, O-CH_2_-CH_2_). 13C NMR (75 MHz, CDCl_3_): δ = 189.3, 145.2, 140.2, 135.1, 133.2, 129.1, 128.9, 127.4, 113.5, 111.1, 109.5, 67.3, 66.9, 65.3, 62.5, 59.6 (36 C). MS (EI, 70 eV): m/z (%) = 592 (16) [M]+. C_36_H_36_N_2_O_6_ (592.69). Calcd: C 72.95; H 6.12; N 4.73; found: C 72.84; H 6.07; N 4.60.

### Synthesis of N,N`-Dicarboxymethyl Phenhomazine (**9)**

Triethylamine (1.0 ml) was added dropwisely to a refluxing mixture of tetrahydrophenhomazine **7** (0.356 g, 0.001 mol), and bromoacetic acid (0.278 g, 0.002 mol) in ethanol (100 ml). The mixture was refluxed for 2 h. At the end of the reaction as indicated by TLC, the reaction mixture was evaporated under vacuum. The solid formed was collected and recrystallized from ethanol to give **9**. Yield 70.3%, m.p. 210–212 °C. IR (film): ν = 3400−2800, 1710, 1706, 1210, 1195 cm^−1^. 1H NMR (300 MHz, CDCl_3_): δ = 12.5 (s, 2H, COOH), 6.64 (d, 2H, J = 8.58 Hz, Ar-H), 6.56 (dd, 2H, J_1_ = 2.62 Hz, J_2_ = 8.58 Hz, Ar-H), 6.39 (d, 2H, J = 2,62 Hz, Ar-H), 4.54 (d, 2H, J = 13.8 Hz, Ar-CH_2_-N), 4.36 (d, 2H, J = 13.8Hz, Ar-CH_2_-N), 4.20 (s, 4H, N-CH_2_-CO-), 4.28-4.20 (m, 2H, O-CH_2_-CH_2_), 4.03-3.95 (m, 2H, O-CH_2_-CH_2_), 3.62−3.44 (m, 4H, O-CH_2_-CH_2_), 3.37−3.16 (m, 2H, O-CH_2_-CH_2_), 2.99−2.82 (m, 2H, O-CH_2_-CH_2_). 13C NMR (75 MHz, CDCl_3_): δ = 175.3, 147.2, 145.3, 140.9, 125.5, 113.6, 109.9, 107.5, 68.3, 67.8, 67.1, 62.9 (24 C). MS (EI, 70 eV): m/z (%) = 472 (24) [M]^+^. C_24_H_28_N_2_O_8_ (472.18). Calcd: C 61.01; H 5.97; N 5.93; found: C 60.90; H 5.85; N 5.81.

### Synthesis of N,N`-Diethoxycarbonylmethyl Phenhomazine (**10)**

To a refluxing mixture of tetrahydrophenhomazine **7** (0.356 g, 0.001 mol), and ethyl bromoacetate (0.334 g, 0.002 mol) in ethanol (100 ml), triethylamine (1.0 ml) was added dropwisely. The reflux was continued for 2 h. The reaction was followed up by TLC. At the end, the reaction mixture was evaporated under vacuum. The solid obtained was collected and recrystallized from ethanol to give **10**. Yield 77.6%, m.p. 199–201°C. IR (film): ν = 1732, 1729, 1245, 1187 cm^−1^. 1H NMR (300 MHz, CDCl_3_): δ = 6.65 (d, 2H, J = 8.58 Hz, Ar-H), 6.58 (dd, 2H, J_1_ = 2.64 Hz, J_2_ = 8.58 Hz, Ar-H), 6.40 (d, 2H, J = 2,64 Hz, Ar-H), 5.54 (d, 2H, J = 14 Hz, Ar-CH_2_-N), 4.25 (s, 4H, N-CH_2_-CO-), 4.36 (d, 2H, J = 14 Hz, Ar-CH_2_-N), 4.28-4.20 (m, 2H, O-CH_2_-CH_2_), 4.10 (q, 4H, J = 8 Hz, CH_3_-CH_2_-CO-), 4.03-3.95 (m, 2H, O-CH_2_-CH_2_), 3.62−3.44 (m, 4H, O-CH_2_-CH_2_), 3.37-3.16 (m, 2H, O-CH_2_-CH_2_), 2.99-2.82 (m, 2H, O-CH2-CH2), 1.11 (t, 6H, J = 8 Hz, CH3-CH2-CO-). 13C NMR (75 MHz, CDCl_3_): δ = 170.1, 147.2, 142.1, 125.5, 112.3, 109.3, 107.7, 68.3, 67.8, 67.3, 61.0, 60.6, 58.5, 14.2 (28 C). MS (EI, 70 eV): m/z (%) = 528 (22) [M]^+^. C_28_H_36_N_2_O_8_ (528.25). Calcd: C 63.62; H 6.86; N 5.30; found: C 63.31; H 6.18; N 4.93.

### Anti-Cancer Screening

The newly synthesized derivatives were assessed against three cancer cell lines; namely hepatocellular carcinoma (HepG-2), breast adenocarcinoma (MCF-7) and Colon Carcinoma (HCT-116) using standard MTT assay (El-Faham, et al., 2014; Elsayed, et al., 2016; Amr, et al., 2018). The assay depends on the mitochondrial reduction of yellow MTT (3-(4,5-dimethylthiazol-2-yl)-2,5-diphenyltetrazolium bromide) to purple formazan. Briefly, cells were propagated in RPMI-1640 medium supplemented with 1% antibiotic–antimycotic mixture and 1% L-glutamine at 37°C and 5% CO2. Upon investigation, cells were plated at a concentration of 104 cells/well in 96-well microtiter plates and incubated for 24 h. Accordingly, exhausted medium was aspirated and fresh medium was added. Then cells were treated with different concentrations of the prepared compounds (0.78–100.00 μg/ml), and inmcubation proceeded for another 48 h. Afterwards, medium was aspirated and 40 μl MTT (2.5 mg/ml/well) was added and the plates were futher incubated for 4 h. The reaction as terminated by the addition of 200 μl/well of 10% sodium dodecyl sulfate, and the plates were incubated overnight to dissolve the developed formazan dey. Doxorubicin was used as positive control. DMSO is the vehicle used for dissolution of compounds, and its final concentration on the cells was less than 0.2%. Absorbance was read using a microplate multi-well reader at 595/620 nm. Results were statistically evalued using an independent *t* test and SPSS 11 program. The percentage inhibition in cell viability was calculated:

(Absorbanceextract/AbsorbanceDMSO)−1 × 100%

A probit analysis was carried for IC50 and IC90 determination using the SPSS 11 program.

## Results and Discussion

### Synthesis

**SCHEME 1:**
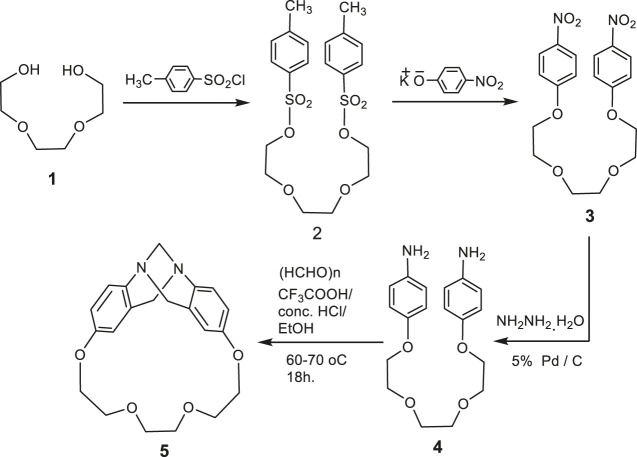
Synthetic rout for trögerophane 5.

In the present study, we synthesized some newly phenhomazine derivatives, **6–10**, using 1,4,7,10-tetraoxa[10](2,8)trögerophane, **5** as a starting material. The rigidity, V-shape, and the presence of a C2 axis of symmetry, have imparted unique structural features on the molecule. Including crown ether oxygens was thought to provide the macrocycle with many important and interesting characteristics. Accordingly, oxygen atoms can increase the solubility in organic solvents by taking advantage of the flexibility of the ether linkage. Also, crown ether linkage can provide the macrocyle with complexing capabilities. In addition, it can keep the chirality of the molecule unchanged when the endomethylene bridge is removed. Therefore, increasing the energy barrier against inversion of the dibenzodiazocine moiety will produce in one fixed conformation for this macrocycle. Earlier, we have reported the synthesis of this trögerophane 5 (Ibrahim, et al., 1998; Miyahara, et al., 1999). In this work we aimed at improving the method used to synthesize this compound, in addition to introduce an unprecedented study about the biological activities of **5** with some of its precursors and its newly synthesized phenhomazine derivatives. The requisite precursors for the cyclization were synthesized via simple processes. Heating triethylene glycol bis(p-toluenesulfonate) **2** with p-nitrophenoxide afforded the corresponding bis(p-nitrophenyl)ether 3, which was treated with hydrazine hydrate in the presence of 5% Pd/C as catalyst to give bis(p-aminophenyl)ether **4** (Ibrahim, et al., 1998; Miyahara, et al., 1999). In the reported method, the intramolecular condensation cyclization of 4 to form 5 in 45% yield, was carried out by reaction with 37% formalin in the presence of concentrated hydrochloric acid under moderately dilute conditions in ethanol at room temperature for 13 days. In order to reduce longer reaction time (13 days) as well as higher solvent consumption, a wide variety of reaction conditions were searched by analyzing the products via HPLC. From the modified method, it can be concluded that raising the reaction temperature to 60–70 °C and using TFA with conc. HCl, and replacing formalin with p-formaldehyde, shortens the reaction time to 18 h, as well as decreasing the amount of consumed ethanol without significantly affecting the yields of trögerophane **5** (43%) (Scheme 1).

**SCHEME 2:**
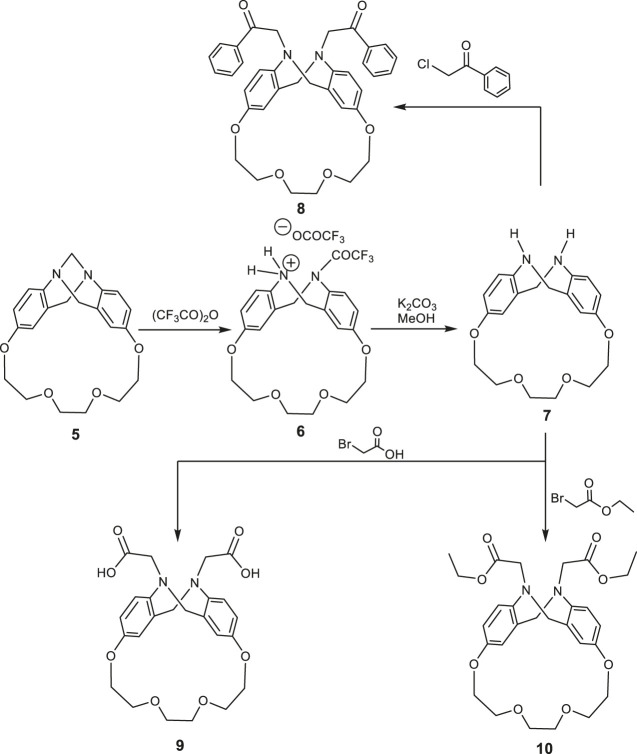
Synthetic routs for phenhomazine derivatives 6–10.

Treatment of trögerophane **5** with refluxing trifluroacetic anhydride afforded the N-trifluroacetamide phenhomazine trifluroacetate **6**, which was stirred with potassium carbonate in methanol for 3 h to afford a good yield of 1,4,7,10-tetraoxa[10](2,8)-5,6,11,12-tetrahydrophenhomazine **7** (Scheme 2). The structures of both **6** and **7** were confirmed on the basis of their elemental and spectral data. The IR spectrum of **7** reveals only one absorption band for the N-H stretching at ν 3398 cm^−1^ indicating the symmetry of the molecule and the absence of intramolecular hydrogen bonding between the secondary amine protons and the transanular nitrogen. 1H-NMR spectrum showing two N-H protons appearing as broad singlet at δ 3.96–3.72 ppm is consistent with the assumed. Reaction of tetrahydrophenhomazine **7** with active electrophiles, namely phenacyl chloride, bromoacetic acid, and ethyl bromoacetate, in the presence of triethyl amine under reflux, afforded the corresponding N,N`-disubstituted phenhomazines **8, 9** and **10**, respectively (Scheme 2).

The IR spectrum of N, N`-diphenacyl phenhomazine **8** revealed two bands at ν 1698, 1694 cm^−1^ indicating the phenacyl C=O bonds. In case of the N,N`-dicarboxymethyl phenhomazine **9**, a broad band at ν 3400–2800 cm^−1^ was clearly indicating that hydrogen bonded carboxylic O-H, and others at ν 1710, 1706 cm^−1^ were due to the C=O of the carboxylic group. Furthermore, N,N`-diethoxycarbonylmethyl phenhomazine **10**, gave bands at ν 1732, 1729 cm^−1^ which can be related to the ester C=O bonds. In addition to these IR data, the absence of the stretching vibration band of the N-H of the tetrahydrophenhomazine, proved that the electrophylic substitution reaction has occurred as expected. In addition to their 13C-NMR, which were consistent with the proposed structures of **8, 9,** and **10**, their 1H NMR spectra revealed highly indicative peaks which supported the claimed structures. For example, the singlet at δ 4.5 ppm indicated 4 protons of the two methylene groups of the phenacyl moiety. The singlet at δ 12.5 ppm in the 1H NMR spectrum of **9** is quiet enough to confirm the presence of the carboxylic protons. Finally, the presence of a quartet at δ 4.10 ppm, and a triplet at δ 1.11 ppm with the same coupling constant (J = 8 Hz), proved the presence of the ethyl group protons in the structure of **10**.

### Anticancer Activity

**FIGURE 2:**
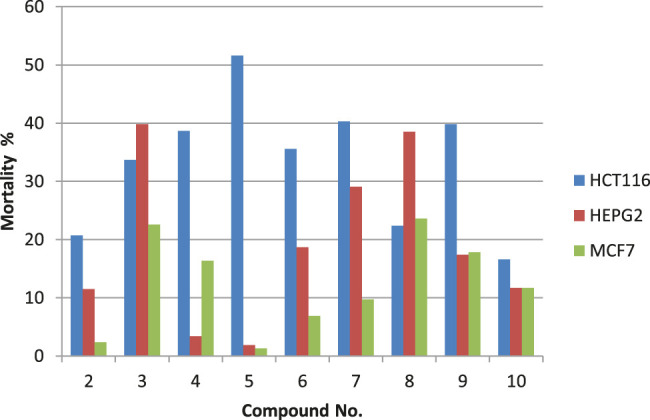
Percent mortality values for the prepared compounds against different cancer cell lines tested at 100 μg/ml.

The cytotoxic potentials of the prepared compounds **2–10** against the investigated human tumor cell lines HCT-116, HepG-2 and MCF-7) were investigated using standard MTT assay with doxorubicin as the reference drug. DMSO was used as the negative control. Cells were exposed to different concentrations of the prepared compounds (0.78–100.00 μg/ml). Results presented in Figure 2 show that only trögerophane **5** had promising cytotoxic effects against HCT-116 carcinoma cell, where the obtained with IC_50_ recorded 92.7 μg/ml. Other tested compounds showed some sort of weak cytotoxicity, however, the investigated concentration range was not appropriate to obtain IC_50_ values for these compounds. Results also revealed that the obtained IC_50_ value for the most potent compound, **5**, against HCT-116, was almost similar to that obtained by the tested positive drug, doxorubicin (85.3 μg/ml).

Results showed that different synthesized derivative reacted differently towards various cell lines. This is expected, since different cell types react differently towards affecting compounds due to their inherent differences in membrane structures and functions (Kaplánek et al., 2015; Elsayed, et al., 2016; Amr, et al., 2018). Furthermore, our results revealed that the prepared trögerophane **5** had showed the most promising cytotoxic effect against colon cancer cell line. This is in good agreement with those results published earlier, where Gaslonde et al. (2011) reported the cytotoxic potentials of their prepared trögerophanes against L1210 leukemia and KB-3-1 solid tumor cell lines. They also found that the prepared trögerophane were more potent than their corresponding parent molecules. Furthermore, Kaplánek et al. (2015) synthesized and evaluated the anticancer potentials of different hydrazone derivatives prepared based on tröger’s base structure. They also reported promising cytotoxic potentials against various cancer cell lines ranging from 0.05 to >100 μM. However, it is difficult to compare our obtained IC_50_ values with those previously reported in the literature against the same cell line due to different descriptors of activities, i.e IC_50_, EC_50_, GI_50_, etc.), different structures, as well as different experimental conditions.

## Data Availability

The original contributions presented in the study are included in the article/Supplementary Material, further inquiries can be directed to the corresponding author.
